# Technology we need to think differently to survive

**Published:** 2012-06-15

**Authors:** George Crooks

**Affiliations:** Scottish Government Health Department, Edinburgh, Scotland

**Keywords:** national telehealth service, Scotland, digital channels, long-term care

## Abstract

The financial climate coupled with the ageing population and the effects of increasing long-term conditions lead to one inevitable outcome for health and care systems. The number of people requiring care will increase significantly, whereas the number able to deliver care (both formal and informal carers) will reduce. Is this a challenge or an opportunity?

The problem is not insoluble, moving to a health and care model, redesigned so that service users are empowered to not simply participate in their own care but deliver their own care, supported by informal carers cannot simply deliver efficiencies but deliver services sustainable into the future. This is supported by the independent evaluation of the national telecare service in Scotland.

Scotland has a national telehealth strategy. Key is the use of digital channels to deliver care services, creating capacity in face to face care services. Scotland has committed significant resources (£80 M) in 2012/2013 to support reshaping care including the deployment of supported technologies into patients homes and has invested significantly in accredited education and training programmes.

The key to success is using technologies already present in peoples homes in an innovative way. Solutions that grow with the user delivering health, care and wellbeing benefits.

